# Influence of different finishing, aging with coffee, and repolishing protocols on the properties of nanoparticle composite resins

**DOI:** 10.4317/jced.61653

**Published:** 2024-06-01

**Authors:** Renata-de Paula Vargas, Alexandre-Coelho Machado, Gisele-Rodrigues da Silva, Amanda-de Souza Miranda, Murilo-Guimarães Campolina, Paulo-César-Freitas Santos-Filho, Murilo-de Sousa Menezes

**Affiliations:** 1 DDS, MS, Graduate student. Post-Graduate Program in Dentistry, School of Dentistry, Federal University of Uberlândia, Uberlândia, Minas Gerais, Brazil; 2 DDS, MS, PhD. Professor. Basic, Technical, and Technological Education, Technical School of Health, Federal University of Uberlândia, Uberlândia, Minas Gerais, Brazil; 3 DDS, MS, PhD. Professor. Division of Operative Dentistry and Dental Materials, School of Dentistry, Federal University of Uberlândia, Uberlândia, Minas Gerais, Brazil; 4 Graduate student. School of Dentistry, Federal University of Uberlândia, Uberlândia, Minas Gerais, Brazil; 5 MS, Graduate student. Post-Graduate Program in Dentistry, School of Dentistry, Federal University of Uberlândia, Uberlândia, Minas Gerais, Brazil; 6 DDS, MS, PhD. Professor. Division of Operative Dentistry and Dental Materials, School of Dentistry, Federal University of Uberlândia, Uberlândia, Minas Gerais, Brazil; 7 DDS, MS, PhD. Professor. Division of Operative Dentistry and Dental Materials, School of Dentistry, Federal University of Uberlândia, Uberlândia, Minas Gerais, Brazil

## Abstract

**Background:**

Considering the variability of finishing protocols for composite resins, the literature does not offer a consensus about the influence of these approaches to obtain a final polishing and whether the physical properties of these composite resins change at different analysis times. Therefore, the study analyzed the microhardness, roughness, color stability, and gloss of a nanocomposite resin with different finishing, aging with coffee, and repolishing protocols.

**Material and Methods:**

Nanocomposite resin samples were divided into three finishing protocol groups: Diamond burs (F and FF), multi-fluted tungsten carbide burs (18 and 30 flutes), and coarse and medium abrasive discs (Soflex-3M). All protocols used spiral rubber tips (F and FF) for polishing. Knoop microhardness (KHN), roughness (Ra), color changes (ΔE00 and YI), and gloss (GU) were analyzed. Scanning electron microscopy provided images of resins and finishing and polishing instruments.

**Results:**

Resin KHN (*p*<0.001) decreased, and Ra (*p*<0.001), ΔE00 (*p*<0.001), and YI (*p*<0.001) increased after aging with coffee, regardless of finishing protocol. Abrasive discs showed lower color changes, YI, and Ra and higher GU. Repolishing restored KHN and Ra but not ΔE00 (*p*>0.05) and YI (*p*>0.05).

**Conclusions:**

Abrasive disc finishing reduced roughness and yellowness and increased nanocomposite resin gloss after aging with coffee.

** Key words:**Color, Composite resins, Dental materials, Staining, Surface properties.

## Introduction

Composite resin restorations feature among the more popular procedures performed by dentists in clinical practice (1), as they provide an excellent treatment alternative for the most common dental problems, such as carious and non-carious lesions and dental fractures, by reestablishing function and esthetics of altered and missing dental structures. Additionally, they offer low costs, excellent physical and mechanical properties, and high dental biomimetic capabilities due to various colors, opacity levels, and nanotechnology that proposes functional and esthetic improvements (2).

Composite resin restorations may achieve long-term success due to various patient-related factors, such as parafunctional habits (3), diet habits (4,5) and limited mouth opening (6). During the restorative procedure, these factors may refer to the operative technique, such as professional experience, saliva contamination (7), curing time and quality,8 and improper use of infection control barriers in light-curing devices (9). Also, finishing and polishing are crucial clinical steps for completing composite resin restorations (10).

Finishing improves the anatomical shape and removes excesses (11). Polishing follows finishing, providing surface smoothness and brightness that results in a more natural appearance, mimicking the tooth’s characteristics (12). It is worth noting that the failure or absence of this clinical step can cause several problems, such as higher susceptibility to staining (13), biofilm accumulation, and gingival inflammation (14-17). Various finishing and polishing protocols are available, involving diverse protocols, instrument types, and commercial brands. These methods include multi-fluted tungsten carbide burs, diamond burs (18), abrasive discs, and rubber instruments with different abrasiveness, shapes, and compositions (aluminum oxide-based or diamond-based). This variety can sometimes confuse dentists in their daily dental office routine (19).

Resin surface roughness may accumulate more bacterial plaque, reduce restoration durability, and cause problems such as deterioration, brightness reduction, and color changes (20). Microhardness is relevant for predicting material wear resistance from the chewing process of patients (21,22). Color stability is critical for composite resin restorations (23). Laboratory studies can assess color changes using the CIE Lab system, which evaluates color perception and acceptability in clinical and social settings (24). Visual color difference thresholds may help evaluate clinical performance and experimental findings on dental materials (25). Consuming acidic foods and beverages or those with coloring agents may alter color stability and surface morphology in composite resins (26,27). Coffee is a frequently consumed beverage (28) with a high staining/degradation capacity on composite resins due to its pigmentation, high temperature, and acidity (29), potentially compromising restoration longevity (30).

Superficial stains in aged restorations may occasionally be removed by repolishing (31,32) a minimally invasive clinical procedure to remove extrinsic discoloration from restoration surfaces and promote adequate smoothness (31). It contributes to the maintenance and longevity of composite resin restorations, reducing the need for replacements (33).

The current literature does not explain whether the finishing procedure affects the polishing ability of composite resin restoration, interfering with their physical properties. In this context, it is worth investigating the surface microhardness, roughness, gloss, and color stability of nanoparticle composite resins associated with different finishing methods. Therefore, this study hypothesized that A) different finishing protocols affect the physical properties of nanoparticle composite resins after aging with coffee and B) repolishing may restore the resin composite characteristics achieved after polishing.

## Material and Methods

In this investigation, no humans, animals, or any portion of either were used. Consequently, there was no requirement for research ethics committee evaluation.

-Specimen preparation

Thirty-seven disc specimens (8 mm x 2 mm) were prepared using a nanoparticle composite resin (Z350-3M XT) of A1E shade and divided into three finishing protocol groups (n=11) and SEM (n=4).  Table 1 describes the materials used in this study. The resin was placed into a polytetrafluoroethylene (PTFE) mold using a single-increment protocol. Then, a Mylar strip and a glass plate were positioned on the specimen to ensure a flat surface. The plate was removed after 20 seconds, and the resin was light-cured directly on the Mylar strip. Specimen light-curing followed the manufacturer’s recommendations and used an LED-curing device (Valo, Ultradent, South Jordan, Utah, USA) with light intensity in the standard power mode of approximately 1000 mW/cm2. Irradiance power (mW) and emission spectrum (mW/nm) were measured with an integrating sphere connected to a fiber-optic spectroradiometer (34), ensuring polymerization standardization.

The specimens were stored for 24 hours in an incubator (Solab, Piracicaba, São Paulo, Brazil) and immersed in deionized water at 37.7ºC. The specimens were ground flat with 320-grit silicon carbide paper (3M, Sumaré, São Paulo, Brazil)10 for 15 seconds with water to standardize the initial surface roughness of the composite resin. Subsequently, they were cleaned in an ultrasonic cleaner (Thornton, Vinhedo, São Paulo, Brazil) with deionized water for 10 minutes.

-Finishing, polishing, and repolishing protocols

**Figure 1:**
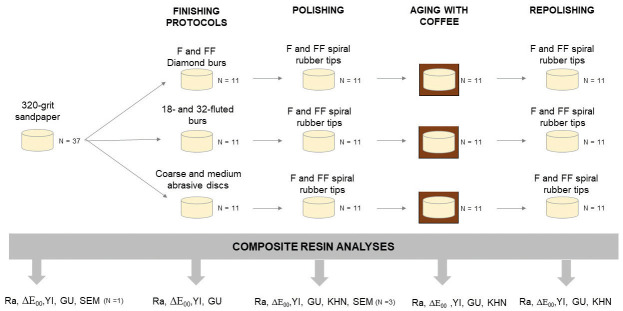
Schematic illustration of the experimental design.

After creating the specimens, the composite resin discs were randomly divided into parallel groups using “https://www.random.org/.” The specimens were categorized (Fig. 1) according to the following finishing protocols: Diamond burs – a fine-grit diamond tip (#2135F, Prima Dental by Angelus) associated with an extra-fine-grit diamond tip (#2135FF, Prima Dental by Angelus); Multi-fluted tungsten carbide burs – an 18-fluted bur (#218, Prima Dental by Angelus) followed by a 30-fluted-bur (#9642, Prima Dental by Angelus); Abrasive discs - a sequence of coarse and medium abrasive discs (Soflex-3M).

A single pre-calibrated operator performed the finishing procedures with controlled speed and water-cooling (Kavo Dental, Joinville, Santa Catarina, Brazil). Each instrument received gentle hand pressure in various directions (10) for 20 seconds (11). The instruments were not used more than five times on the specimens.10 All groups were polished with F and FF spiral rubber tips at low speed and water-cooled for 20 seconds in each instrument. After coffee staining, the specimens were repolished using the same polishing tools.

-Staining and aging protocols

After polishing, the specimens were immersed in coffee for the staining protocol. A solution was prepared by dissolving 3.0 g of coffee (Nescafé Original, Nestlé, São Paulo, Brazil) in 150 ml of deionized water at 92°C (35). The coffee manufacturer provided the applied temperature and dilution. The resulting solution was stored in 2.0ml Eppendorf tubes. The specimens remained immersed in this solution for seven consecutive days, with a daily solution exchange, simulating two years of aging (35,36).

After storing the specimens in an incubator (Solab, Piracicaba, São Paulo, Brazil) for seven days at 37.7°C, they were washed with deionized water for five seconds, placed in an ultrasonic cleaner (Thornton, Vinhedo, São Paulo, Brazil) for 10 minutes, and dried.

-Knoop microhardness measurement

Knoop microhardness values were measured at three evaluation moments: after polishing, after staining, and after repolishing. A microhardness tester (FM-7000, FUTURE-TECH CORP, Kawasaki, Japan) connected to software for Windows provided the measurements. The used indenter was a pyramidal diamond with a square base, forming a rhombus, which characterizes the Knoop microhardness analysis.

The surface of each specimen received indentations in five areas near the center of the sample. The test used controlled force, applying a 50g (0.98N) load for 15 seconds in each indentation. The statistical calculations considered the mean of the described measurements.

-Surface roughness measurement

Roughness was analyzed at the five mentioned evaluation moments: baseline, after finishing, after polishing, after staining, and after repolishing. The specimens were stabilized on an acrylic resin device attached to the machine’s support. The test used a contact profilometer (Mitutoyo, Aurora, IL, USA). Mean roughness (Ra) was measured with a static load of 5 N and a speed of 0.05 mm/s. The cut-off was 0.25 μm in a sequential mode, and the measurement distance was 1 mm. Three readings were made for each specimen from the surface center, and the arithmetic mean was calculated.

-Color analysis

A sphere spectrophotometer (Ci64UV, X-Rite, Grand Rapids, MI, USA) measured the color of each sample, and the color system by the Commission Internationale de L’Eclairage (CIE) based on color dimensions measured by L* (white/black axis), a* (red/green axis), and b* (yellow/blue axis) evaluated color changes. These measurements occurred at different times: initial and after finishing, polishing, coffee staining, and repolishing.

The specimens were placed on a standardized metal support for color readings, constantly evaluating the same area (4mm diameter). A standard illuminant D65 took the measurements with a wavelength from 400 to 700 nm and with specular included (SPIN mode). The spherical shape of the spectrophotometer made the object diffusely illuminated, and the detector received the reflected light at 88º to the resin surface. Color was measured in triplicate against a white background (L* white = 95.2, a* white = 21.2, b* white = 50.3), and the mean values aided data analysis.

The yellowness index (YI) calculation by the ASTM E313 method used the formula YI = (100(CxX - CzZ)) / Y, where X, Y, and Z are the coordinates of the CIE Tristimulus values. Total color change considered the initial analysis using the CIEDE2000 formula ΔE00 = [(ΔL/KL SL)2 + (ΔC/KCSC)2 + (ΔH/KHSH)2 + RT (ΔC/KCSC) (ΔH/KHSH)]1/2, where ΔL, ΔC, and ΔH are lightness, chroma, and hue differences between color measurements; KL, KC, and KH are the parametric factors for viewing conditions and influence of illuminating settings; RT is the interaction of hue and chroma differences in the blue region; SL, SC, and SH are weighing functions for color difference adjustments considering the location variation of L*, a*, and b* coordinates (37).

Color differences were compared with baseline data (after sample fabrication) after finishing and polishing, with finishing and polishing data after staining, and with stained data after repolishing.

-Gloss assessment

A small-area glossmeter (3NH Global, NHG60M, Shenzhen, China) measured gloss. A custom-made, 10-mm-thick, black polytetrafluoroethylene mold was placed over the specimen during measurements to allow accurate specimen positioning and eliminate the influence of the overhead light. A 60° angle was applied to evaluate the gloss at the center of the sample. Gloss measurements were expressed in gloss units (GU).

-Scanning electron microscope (SEM)

The VEGA 3 (TESCAN) scanning electron microscope (SEM), with an acceleration voltage of 5kV, provided representative images of the instruments used in this study (finishing tools at 100X and polishing tools at 300X). Moreover, representative images of the resin surface were captured immediately after fabrication and finishing and polishing procedures at 1.00KX magnification.

-Statistical analysis

The results were organized into Tables using Microsoft Office Excel and subsequently exported to the Jamovi 2.0 statistical analysis program (dev.jamovi.org). Repeated-measures ANOVA and Tukey’s test compared the color parameters (ΔE00, YI, and gloss), roughness (Ra), and microhardness (KHN). The assessment time was the repeated factor. All analyses occurred at a 95% significance level (α=0.05).

## Results

 Table 2 presents KHN,  Table 3 GU and Ra, while  Table 4 presents YI and ΔE00.

The groups did not show statistical differences after polishing for microhardness (*p*>0.05), ΔE00 (*p*>0.05), and YI (*p*>0.05), while groups finished with abrasive discs showed lower Ra and higher GU.

Resin microhardness (*p*<0.001) decreased, and Ra (*p*<0.001), ΔE00 (*p*<0.001), and YI (*p*<0.001) increased after coffee staining, regardless of the finishing protocol. The abrasive disc groups showed higher gloss values.

Repolishing restored the original composite resin’s microhardness and roughness, regardless of the finishing protocol. The same did not occur for the color (*p*>0.05) obtained after the initial polishing, even though yellowness decreased.

**Figure 2:**
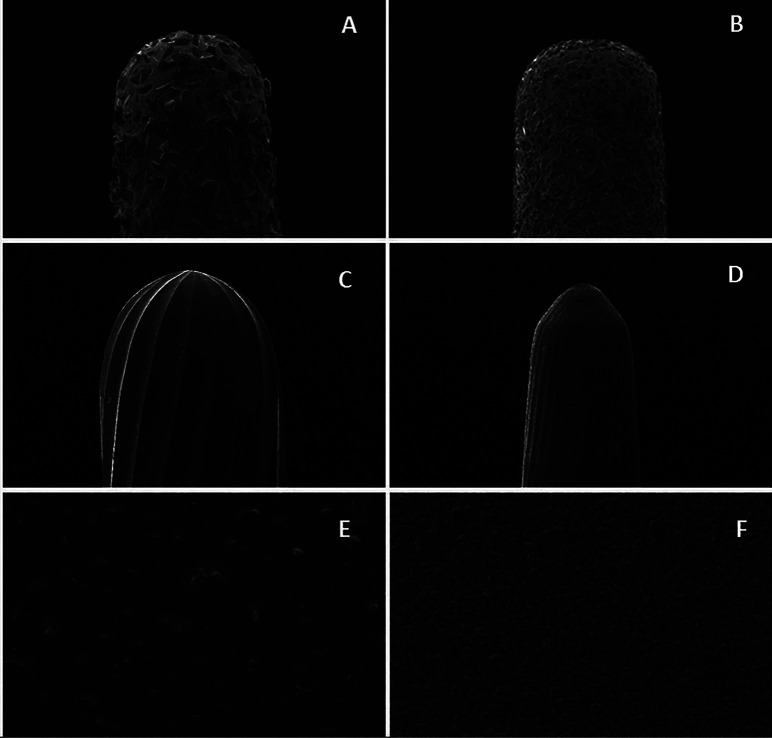
Morphological images of rotary finishing instruments at 100x. A) Diamond bur - fine-grit diamond tip (#2135F). B) Diamond bur – extra fine-grit diamond tip (#2135FF). C) Multi-fluted tungsten carbide bur - 18-fluted bur (#218). D) Multi-fluted tungsten carbide bur - 30-fluted bur (#9642). E) Abrasive discs – coarse. F) Abrasive discs – medium.

**Figure 3:**
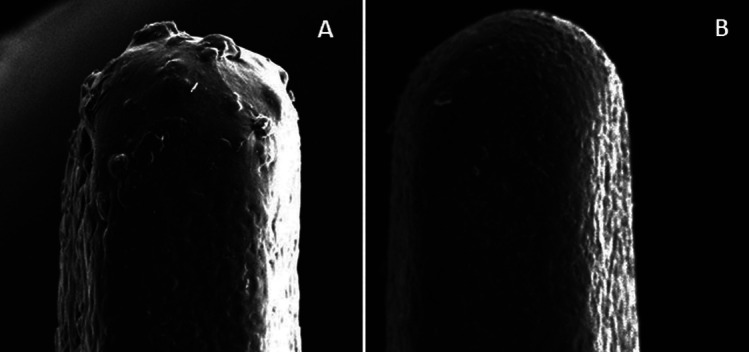
Morphological images of rotary polishing instruments at 300x.

Figure 2 shows the SEM image of the different finishing rotary instruments used in the study, and Figure 3 presents the polishing tools. Morphology changed, showing milling cutters for multi-fluted burs and diamond granules of different sizes and arrangements in the diamond tips.

**Figure 4:**
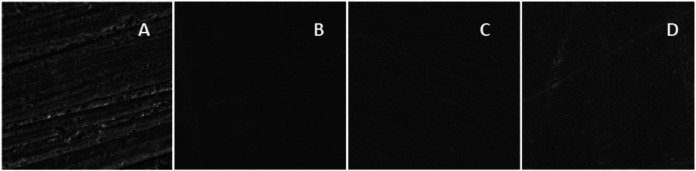
Morphological images of the resin surface at 1.00KX. A) Baseline. B) Finishing with diamond tips and polishing. C) Finishing with multi-fluted burs and polishing. D) Finishing with abrasive discs and polishing.

Figure 4 shows the composite resin surface morphology under different finishing protocols. Finishing with multi-fluted burs caused more surface irregularities.

## Discussion

The finishing protocols used in this study affected the physical properties of the nanoparticle composite resin immersed in coffee, especially regarding surface roughness and color, confirming the first hypothesis. Overall, finishing composite resin restorations with discs with decreasing abrasiveness promoted a smoother surface with higher color stability and gloss. Achieving the final surface smoothness improved the esthetic perception and reduced the chances of bacterial plaque accumulation, recurrent caries, and restoration discoloration (13,17).

Composite resins exposed to the oral environment are susceptible to aging from colored acidic foods and beverages, such as coffee (26), one of the most consumed beverages worldwide, second only to water (28). Coffee intake is usually daily and at high temperatures (29); hence, investigating the behavior of this beverage was relevant considering the variability of finishing protocols.

As for the perceptibility and acceptability thresholds (25), the simulated coffee staining caused unacceptable color changes perceptible to the human eye, regardless of the finishing protocol. Furthermore, YI increased, probably due to the adsorption and absorption of yellow dyes from coffee, which have low polarity and penetrate deeper layers of the composite resin. Repolishing helped to decrease the staining but not to restore the initial resin color, rejecting the second hypothesis. The consequence of this event in the clinical routine of dentists is patient dissatisfaction due to long-term and potentially irreversible esthetic compromises. Hence, patients should be informed about the color stability of composite resins and the potential impact of diet habits, as coffee consumption may cause continuous color changes over time (30).

Regarding surface roughness, the study simulated an extremely rough surface onset, which may clinically occur after occlusal restoration adjustments in the oral cavity. Therefore, the surface was standardized following a previous protocol (10) using 320µm-grit silicon carbide sandpaper. The specimens were finished after surface standardization, showing that multi-fluted burs and diamond tips produced similar roughness in the composite resin surface, higher than after using abrasive discs. The scanning electron microscopy (SEM) images and manufacturer’s information demonstrated that diamond tips used in the study had a particle size from 38 to 64 µm for fine (F) grit and 0 to 38 µm for extra-fine (FF) grit. The F tips presented larger and more spaced particles, and the FF tips had smaller and closer particles, as expected. The 18- and 30-fluted burs created more substantial irregularities on the composite resin surface, justifying the higher roughness values of this finishing protocol. However, the two finishing methods did not differ after aging with coffee. That may be due to the low pH (4.9) of coffee, which might have superficially dissolved the composite resin, thus equalizing its surface roughness after finishing with multi-fluted burs and diamond tips. Furthermore, roughness values after repolishing were lower for the discs and similar between diamond tips and multi-fluted burs.

Choosing a finishing tool requires more than the analysis in this study because its use highly depends on restoration location and size. Composite resin microhardness refers to material resistance to definitive deformation when subjected to penetration involving complex forces and stresses (21). This study did not show statistical differences in microhardness for the finishing protocols applied to the composite resin. That may be due to light-curing performed at adequate timing and using a light-curing device with proper light incidence (24). However, coffee decreased microhardness because it caused topographic irregularities confirmed by higher roughness values. Removing the most superficial layer after repolishing exposes a resin surface unchanged by aging with coffee. Thus, microhardness values after repolishing were similar to those after the finishing and polishing protocols without aging.

Finishing with abrasive discs yielded a surface with lower roughness and higher color stability and gloss. SEM images showed a homogeneous disc surface with small and well-distributed granules, as seen in Figure 2. This morphological characteristic of discs might have been crucial for their effectiveness in the finishing process. Moreover, disc action may have been enhanced because the finishing material was from the same manufacturer of the polishing points used in all groups. The polishing instruments may have been produced to complement the abrasive discs used in this study. However, their clinical application represents a limitation because discs may complicate the maintenance of anatomical characteristics in concave areas, such as lingual/palatal and occlusal surfaces (16). These regions have grooves, scars, and fissures that hinder the access of discs, challenging the excess removal of restorative material and functional and esthetic adjustments in anterior tooth restorations. Therefore, diamond tips or multi-fluted burs will be imperative in the clinical routine at some point.

Clinical studies are essential to confirm whether different finishing methods would impact the survival and maintenance of composite resin restorations.

## Conclusions

The study hypotheses allow the conclusions that:

1) Different finishing protocols may affect the physical properties of nanoparticle composite resins after aging with coffee. Abrasive discs promoted lower roughness and color changes and higher gloss.

2) Repolishing restored the microhardness and roughness values close to those after polishing. However, the same did not occur for the initial color of the composite resin aged with coffee.

## Figures and Tables

**Table 1:** Material compositions obtained from manufacturers’ information and safety data sheets.

Material	Manufacturers	Type/Color	Composition	Batch number
Filtek Z350XT	3M ESPE	Nanofilled/ A1 Enamel	Bis-GMA, Bis-EMA, UDMA, TEGDMA, 82% filler (0.004 to 10 µm - based on silica and zirconia)	2223800378
Fine-grit diamond bur	Prima Dental, Angelus, Londrina, PR, Brazil	2135 F	Diamond cutter	12133
Extra fine-grit diamond bur	Prima Dental, Angelus, Londrina, PR, Brazil	2135 FF	Diamond cutter	8932
Multi-fluted burs 12 flutes	Prima Dental, Angelus, Londrina, PR, Brazil	FG218	Carbide milling cutter	12014BR
Multi-fluted burs 30 flutes	Prima Dental, Angelus, Londrina, PR, Brazil	FG9642	Carbide milling cutter	11812BR
Sof-Lex pop-on discs	3M ESPE, Seefeld, Germany	Abrasive discs/Orange series 4931 G	Polyester film coated with aluminum oxide abrasive and metallic center	2221400365
Sof-Lex pop-on discs	3M ESPE, Seefeld, Germany	Abrasive discs/Orange series 4931 M	Polyester film coated with aluminum oxide abrasive and metallic center	2218600522
Sof-Lex	3M ESPE, St. Paul, MN, USA	Two-step rubber wheel polishing system	Diamond particles impregnated in a thermoplastic elastomer	2207400420

**Table 2:** Mean microhardness (KHN and ± standard deviation) and statistical category with sample repetition – Tukey’s test.

Finishing protocol	Polished				Aging				Repolished			
Diamond burs	82.81	±	0.0481	Aa	80.33	±	0.0603	Ab	82.40	±	0.0319	Aab
Multi-fluted burs	82.96	±	0.0401	Aa	79.73	±	0.0245	Ab	82.64	±	0.0239	Aab
Abrasive discs	83.56	±	0.0612	Aa	80.26	±	0.0262	Ab	81.99	±	0.0512	Aab

* Different lowercase letters indicate statistically significant differences between groups (vertical), and uppercase letters between times (horizontal) (p < 0.05).

**Table 3:** Mean measurements ± standard deviation of gloss (GU) and surface roughness (Ra- in µm) of the composite resin, considering the surface finishing protocols at different evaluation times.

Assessment time		Diamond burs					Multi-fluted burs				Abrasive discs			
Baseline	GU		2.19	±	0.75	Ad	2.33	±	0.51	Ad	2.08	±	0.48	Ad
	Ra		0.496	±	0.007	Ad	0.501	±	0.002	Ad	0.495	±	0.004	Ad
Finished	GU		9.18	±	1.18	Bc	10.60	±	1.83	Bc	15.20	±	2.69	Ac
	Ra		0.345	±	0.005	Bc	0.386	±	0.004	Cc	0.162	±	0.002	Ac
Polished	GU		76.80	±	11.40	Ba	74.20	±	4.00	Ba	78.00	±	5.20	Aa
	Ra		0.052	±	0.002	Ba	0.055	±	0.003	Ba	0.037	±	0.003	Aa
Aging	GU		65.8	±	11.30	Ba	73.2	±	5.13	ABa	80.8	±	8.31	Aa
	Ra		0.072	±	0.003	Bb	0.071	±	0.001	Bb	0.054	±	0.004	Ab
Repolished	GU		78.60	±	8.57	Aa	81.90	±	6.69	Aa	85.80	±	6.72	Aa
	Ra		0.05	±	0,000	Ba	0.05	±	0,000	Ba	0.04	±	0.001	Aa

* Lowercase letters indicate statistical differences in the column (finishing at different evaluation times), and uppercase letters in the row (finishing protocols at a given evaluation time) for each surface property

**Table 4:** Means (± standard deviation) the Yellowness index (YI) and color difference (ΔE00) considering the surface finishing protocols at different evaluation times.

Assessment time	Diamond burs					Multi-fluted burs				Abrasive discs			
Baseline	YI	5.59	±	1.23	^Aa^	6.37	±	0.95	^Aa^	6.71	±	1.15	^Aa^
Polished	YI	5.82	±	1.02	^Aa^	4.91	±	1.4	^Aa^	6.59	±	0.98	^Aa^
	ΔE00	1.01	±	0.41	^Aa^	1.18	±	0.38	^Aa^	1.04	±	0.41	^Aa^
Aging	YI	17.2	±	1.21	^Bc^	17.7	±	0.64	^Bc^	14.7	±	1.18	^Ac^
	ΔE00	5.37	±	0.4	^Bc^	5.22	±	0.37	^Bc^	4.92	±	0.60	^Ac^
Repolished	YI	12.5	±	1.00	^Bb^	12.6	±	1.80	^Bb^	10.3	±	1.00	^Ab^
	ΔE00	3.05	±	0.49	^Bb^	2.98	±	0.48	^Bb^	2.36	±	0.52	^Ab^

* Lowercase letters indicate statistical differences in the columm (finishing at different evaluation times), and uppercase letters in the row (finishing protocols at a given evaluation time) for each color parameter

## Data Availability

The datasets used and/or analyzed during the current study are available from the corresponding author.
